# Unlocking the Bioactive Power of *Alchemilla subcrenata* Buser via Optimized Green Extraction

**DOI:** 10.3390/plants15142161

**Published:** 2026-07-13

**Authors:** Jelena Vuković, Jelena Bajac, Gokhan Zengin, Tatjana Majkić, Lidija Petrović, Milena Terzić

**Affiliations:** 1 Faculty of Technology Novi Sad, University of Novi Sad, Bulevar Cara Lazara 1, 21000 Novi Sad, Serbia; jsimic021@gmail.com (J.V.); ilicj@uns.ac.rs (J.B.); lidijap@uns.ac.rs (L.P.); 2 Science Faculty, Selcuk University, Campus Konya, Konya 42130, Turkey; biyologzengin@gmail.com; 3 Faculty of Sciences, University of Novi Sad, Trg Dositeja Obradovića 3, 21000 Novi Sad, Serbia; tatjana.majkic@dh.uns.ac.rs

**Keywords:** *Alchemilla subcrenata* Buser, microwave-assisted extraction, Box–Behnken design, phenolic bioactives, antioxidant potential, enzyme inhibition

## Abstract

*Alchemilla subcrenata* Buser is a montane *Alchemilla* (Rosaceae) species with established traditional medicinal use but only limited prior phytochemical characterization, restricted to preliminary observations on phenolic content and peroxidase activity. This study reports the first systematic optimisation of microwave-assisted extraction (MAE) conditions for this species using a Box–Behnken design within a Response Surface Methodology (RSM) framework. Three extraction variables, ethanol concentration (30–70%, *v*/*v*), solid-to-liquid ratio (1:10–1:20, *w*/*v*), and extraction time (10–30 min), were simultaneously optimised against twelve response variables encompassing total phenolic and flavonoid contents, six antioxidant assays, and four enzyme inhibitory activities (acetylcholinesterase, butyrylcholinesterase, tyrosinase, and α-amylase). Second-order polynomial models demonstrated good predictive ability for most responses (R^2^ = 0.878–0.971 for eleven of the twelve responses), with the exception of metal chelation, which showed a lower fit (R^2^ = 0.671) and was excluded from optimisation; the multiobjective optimization identified optimal conditions of 60% ethanol, 1:12.5 (*w*/*v*) solid-to-liquid ratio, and 10 min extraction time. Under optimal conditions, MAE delivered exceptional in vitro antioxidant and enzyme inhibitory activities from *A. subcrenata*. LC-MS/MS characterisation, representing the first comprehensive phenolic profiling of this species, identified 28 phenolic compounds (1599.7 μg/g extract), dominated by quercetin-3-O-hexoside, gallic acid, catechin, and kaempferol glycosides; naringenin and aesculetin represent novel phytochemical records for *A. subcrenata*. The abundant presence of quercetin hexosides and hydroxycinnamic acids offers a mechanistic explanation for the broad, multi-target bioactivity observed, positioning *A. subcrenata* as a promising source of bioactive phenolics that warrants further investigation for potential nutraceutical, pharmaceutical, and cosmeceutical applications.

## 1. Introduction

The genus *Alchemilla* L. (Rosaceae), commonly known as lady’s mantle, comprises numerous perennial herbaceous species widely distributed across Europe and parts of Asia. Members of this genus have long been used in traditional medicine for the treatment of wounds, gastrointestinal disturbances, gynecological disorders, and inflammatory conditions, primarily due to their rich content of ellagitannins, flavonoids, and hydroxycinnamic acids [1]. Phytochemical investigations of the most studied representatives, particularly *A. vulgaris* and *A. mollis*, have confirmed dense polyphenol profiles that correlate with pronounced antioxidant, anti-inflammatory, antimicrobial, and enzyme-inhibitory activities [2,3]. However, species-level variation in phytochemical composition within *Alchemilla* is well established, meaning that bioactivity data obtained for one species cannot be reliably extrapolated to others; each species requires independent investigation, a conclusion reinforced by evidence that genotype and climatic conditions jointly shape active-compound profiles in other medicinally important taxa [1,2,4].

Against this background, *Alchemilla subcrenata* Buser represents a particularly important but largely neglected subject of inquiry. Distributed across montane meadows and forest margins from the Alps and the Balkans northwards to Scandinavia and Russia [5], this species has received virtually no systematic phytochemical attention. The sparse available data are limited to preliminary observations on the diurnal dynamics of phenolic compound content and peroxidase activity [6], and a preliminary chromatographic analysis that confirmed the presence of phenolic acids and flavonoids without further characterization or bioactivity assessment [7]. No peer-reviewed studies have reported optimized extraction protocols, comprehensive phenolic profiling, or enzyme inhibitory potential for *A. subcrenata*, a significant knowledge gap given the demonstrated bioactivity of closely related species and the growing interest in underexplored botanical resources for pharmaceutical and nutraceutical applications. Notably, ellagitannins and flavonoids, the dominant compound classes in the *Alchemilla* genus, are well-recognized inhibitors of cholinesterases and glycosidases [2], suggesting that *A. subcrenata* may harbor relevant multi-target bioactive potential that remains entirely unexplored.

Efficient recovery of phenolic compounds depends critically on extraction methodology. Conventional solvent extraction, widely used in earlier *Alchemilla* studies, is associated with several drawbacks, including prolonged extraction times, high solvent consumption, and suboptimal recovery of thermolabile compounds [8]. Microwave-assisted extraction (MAE) offers a compelling green alternative, providing rapid and uniform energy transfer that enhances cell disruption while minimizing thermal degradation of sensitive phytochemicals [9,10]. Since MAE performance is highly sensitive to interactions among solvent composition, solid-to-solvent ratio, and extraction time, systematic multivariate optimization is essential. Response surface methodology (RSM) combined with a Box–Behnken design provides exactly this capability, enabling simultaneous modeling of multiple variables with a minimal number of experimental runs [11,12]. Notably, while RSM has been applied to optimize extraction conditions for *A. vulgaris* using deep eutectic solvents [3], MAE-based optimisation has not been explored for this or any other *Alchemilla* species, a gap that the present study addresses.

The present study was therefore designed to optimize MAE conditions for *A. subcrenata* using a Box–Behnken experimental design, with ethanol concentration, solid-to-solvent ratio, and extraction time as independent variables, and total phenolic content, total flavonoid content, antioxidant capacity (DPPH, ABTS, CUPRAC, FRAP, metal chelation, and phosphomolybdenum), and enzyme inhibitory activities (AChE, BChE, tyrosinase, and α-amylase) as response variables. Extracts obtained under optimized conditions were further subjected to HPLC-based phenolic profiling to identify constituents potentially responsible for the observed bioactivities, providing a rigorous scientific foundation for evaluating *A. subcrenata* as a source of high-value functional phytochemicals. To our knowledge, this represents the first comprehensive MAE/RSM optimisation and LC-MS/MS-based phenolic profiling of *A. subcrenata*, building on the limited preliminary data previously available for this species [6,7].

## 2. Results and Discussion

### 2.1. Total Phenolic and Flavonoid Contents

The total phenolic content (TPC) and total flavonoid content (TFC) of *A. subcrenata* Buser extracts^1^ obtained under different MAE conditions are summarized in Table 1.

TPC values ranged from 44.10 to 72.71 mg GAE/g extract, while TFC values ranged from 19.54 to 40.68 mg RE/g extract, indicating that both parameters were substantially influenced by the extraction conditions. These ranges are broadly consistent with total phenolic and flavonoid contents reported for other widely studied culinary and medicinal herbs [13]. The highest TPC values were consistently observed at intermediate ethanol concentrations (~50%, X1), solid-to-liquid ratios of about 1:15 (X2), and short extraction times (10–20 min, X3), as represented by samples 3, 8, 12, and 14. Flavonoid content followed a similar trend to total phenolics, confirming that flavonoids constitute a significant fraction of the total phenolic pool in *A. subcrenata*. The low standard deviations among replicates indicate the reproducibility and robustness of the MAE process.

**Figure 1:**
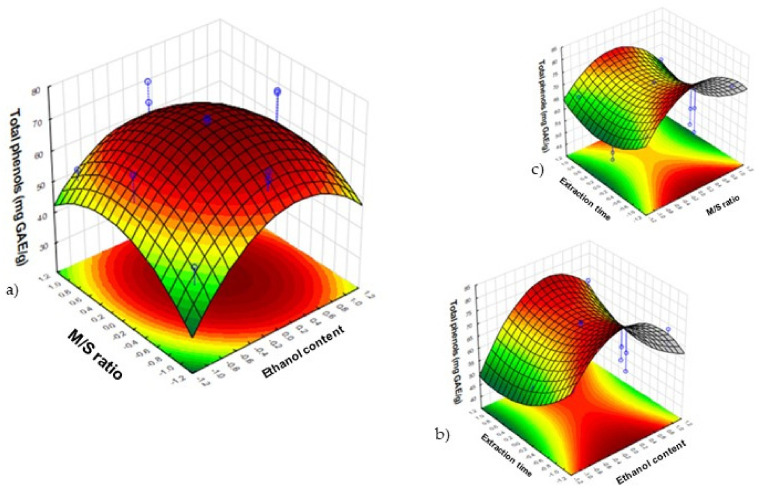
Response surface plots showing the effects of ethanol concentration (X_1_), solid-to-liquid ratio (X_2_), and extraction time (X_3_) on total phenolic content (TPC, mg GAE/g) of *A. subcrenata* Buser extracts: (**a**) X_1_ × X_2_ interaction, (**b**) X_1_ × X_3_ interaction, (**c**) X_2_ × X_3_ interaction.

The three-dimensional response surface plots (Figure 1) illustrate the simultaneous effects of ethanol concentration (X1), solid-to-liquid ratio (X2), and extraction time (X3) on TPC. The second-order polynomial model showed a statistically adequate fit (R^2^ = 0.877), although the lack-of-fit test was significant (Supplementary Table S1), and the overall shape of the response surfaces consistently revealed a well-defined, curvilinear optimum in the multidimensional experimental space.

The response surface depicting the interaction between ethanol concentration and solid-to-liquid ratio (Figure 1a) revealed a pronounced dome-shaped curvature, with TPC reaching its maximum at intermediate ethanol concentrations (~50%) and moderate solid-to-liquid ratios (~1:15). At low ethanol concentrations (<40%), TPC was reduced, reflecting insufficient solubility of moderately polar phenolics, particularly flavonol glycosides, ellagitannins, and hydroxycinnamic acid esters that constitute the major phenolic classes in *Alchemilla* species [1,2]. This effect is more pronounced at high extraction times (Figure 1c). The progressive decline in TPC at ethanol concentrations exceeding 60–70% is consistent with the well-established polarity-window concept in solid–liquid extraction, whereby excessively apolar solvent systems compromise the solubilisation of hydrophilic phenolic glycosides while simultaneously promoting the co-extraction of non-phenolic matrix components, an effect consistently reported when comparing extraction solvents of differing polarity [7,14,15,16]. The response surface illustrating the interaction between ethanol concentration and extraction time (Figure 1b) showed that short extraction times (10–15 min) consistently yielded the highest TPC.

The regression coefficients (β) and coefficients of determination (R^2^) for all fitted quadratic models are summarised in Table 2. For TPC (R^2^ = 0.877), the quadratic effects of ethanol concentration and solid-to-liquid ratio were the dominant significant terms, confirming a curvilinear optimum within the intermediate range of both variables, a pattern also reported for BBD-optimised MAE of *Inula britannica* [12] and *Phyllanthus emblica* [17]. Beyond a certain solvent volume, increasing the solid-to-liquid ratio no longer improves phenolic recovery—a mass-transfer saturation behaviour documented in RSM-guided MAE optimisation studies across diverse plant matrices, including spring onion [18] and coriander seeds [19], and aqueous ethanol extraction of berry leaves and branches [20]. TPC decreased progressively with increasing extraction duration, with prolonged microwave irradiation promoting thermal hydrolysis of hydroxycinnamic acid derivatives and flavonoid glycosides, generating degradation products with reduced Folin–Ciocalteu reactivity [21]. The significant interaction between ethanol concentration and extraction time indicates that at intermediate ethanol levels, moderate extraction times enhanced phenolic recovery [19], further underscoring the necessity of simultaneous multivariate optimisation.

**Figure 2:**
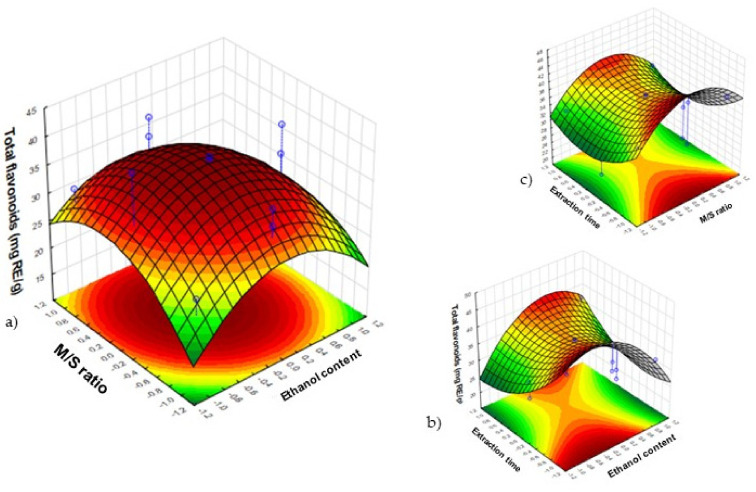
Response surface plots showing the effects of ethanol concentration (X_1_), solid-to-liquid ratio (X_2_), and extraction time (X_3_) on total flavonoid content (TFC, mg RE/g) of *A. subcrenata* Buser extracts: (**a**) X_1_ × X_2_ interaction, (**b**) X_1_ × X_3_ interaction, (**c**) X_2_ × X_3_ interaction.

The response surface plots for TFC are presented in Figure 2 (R^2^ = 0.908; lack-of-fit significant, Supplementary Table S1). TFC values ranged from 19.54 to 40.68 mg RE/g, with the highest value recorded for sample 2 (40.68 mg RE/g; 30% EtOH, 1:15, 10 min) and the lowest for sample 9 (19.54 mg RE/g; 70% EtOH, 1:10, 20 min).

The response surface for TFC exhibited a similar pattern to that for TPC, with maximised values at ethanol concentrations of around 30–50%. The use of either very low or very high ethanol concentrations, combined with a low quantity of material, was not preferable and led to a marked reduction in TFC (with the exception of samples 2 and 13). This behaviour is explained by the predominantly hydrophilic character of flavonoid glycosides in *Alchemilla* species, including quercetin-3-glucuronide, kaempferol glycosides, and luteolin derivatives reported in related species [1,2]. These compounds require more polar extraction media for optimal solubilisation, which accounts for the preference for lower ethanol concentrations, as also observed for *Phyllanthus emblica* [17] and spring onion [18]. At intermediate ethanol concentrations (~50%) and intermediate solid-to-liquid ratios, short extraction times are preferable for higher TFC recovery.

Although extraction time had no statistically significant linear effect on TFC, the interaction between ethanol concentration and extraction time was significant, suggesting a combined effect whereby the influence of one factor varies depending on the level of the other. Their mutual influence indicates that at medium ethanol concentrations and high extraction times, TFC recovery can be improved (Figure 2c), further emphasising the necessity of simultaneous multi-factor optimisation.

ANOVA results confirming the significance of model terms for all twelve responses are provided in Supplementary Table S1.

The TPC and TFC values obtained for *A. subcrenata* under optimised MAE conditions appear higher than previously reported values for *A. vulgaris* obtained by conventional extraction methods (7.17 mg GAE/g and 3.63 mg QE/g dry weight, respectively [22]), and are also higher than values reported for *A. mollis* [23,24] and *A. xanthochlora* [25], confirming both the superior efficiency of MAE and the exceptional phytochemical richness of this previously underexplored species [19,26]. The apparent difference relative to conventionally extracted *A. vulgaris* may reflect two complementary factors: the capacity of microwave-assisted extraction to disrupt plant cell walls more efficiently and shorten extraction time, thereby limiting thermal degradation of labile phenolics [7,8], and the intrinsically rich phenolic composition of *A. subcrenata*, as subsequently confirmed by LC-MS/MS profiling (Section 2.5). It should be noted, however, that these comparisons are approximate, as several literature values are expressed per gram of dry plant material, whereas the present values are expressed per gram of extract; in the absence of extraction-yield-based unit conversion, the magnitude of these differences should be interpreted with caution.

### 2.2. Antioxidant Activity

The antioxidant activities of *A. subcrenata* Buser extracts, assessed across six complementary assays (DPPH, ABTS, CUPRAC, FRAP, metal chelation, and phosphomolybdenum), exhibited pronounced, assay-specific non-linear responses to the three extraction variables (Table 2 and Table 3).

Samples 13 (70% EtOH, 1:15, 30 min), 14 (50% EtOH, 1:10, 10 min), and 15 (70% EtOH, 1:15, 10 min) showed the highest radical-scavenging activities under DPPH and ABTS, as well as the strongest reducing power (CUPRAC and FRAP), with no statistically significant differences among them (Table 3).

Examination of the regression coefficients for DPPH (R^2^ = 0.923) and ABTS (R^2^ = 0.953) reveals that ethanol concentration exerted the dominant positive linear influence on radical scavenging (β_1_ = 58.63 and 218.32, respectively), where increasing the ethanol content significantly increased DPPH and ABTS values. The negative quadratic coefficients (β_11_ = −147.02 and −554.75) indicate that high ethanol concentrations have the opposite effect, contributing to a decrease in DPPH and ABTS values at the upper boundary of X_1_. The DPPH and ABTS values depend on the material-to-solvent ratio in a way that the non-significant linear coefficient implies that the response is not primarily governed by a first-order effect, whereas the significant negative quadratic term indicates a maximum value at medium material-to-solvent ratio. The strong positive quadratic terms for extraction time (β_33_ = 82.85 and 367.49) indicate a curvilinear, U-shaped time response: radical-scavenging activity was highest at the shortest extraction times tested (10 min) and partially recovered at the longest (30 min), with a local minimum at intermediate durations (20 min). This non-monotonic time dependence reflects competing kinetic processes: during the initial phase of microwave irradiation, rapid cell disruption releases intracellular phenolic pools, maximising antioxidant yield; at intermediate times, partial thermal degradation of labile catechol-type structures reduces activity; while at longer durations, extraction of additional thermally stable tannin-derived fragments partially compensates for the loss of monomeric phenolics [7,21].

The CUPRAC (R^2^ = 0.942) and FRAP (R^2^ = 0.962) assays showed dependence on all investigated variables. The CUPRAC value was primarily affected by ethanol concentration and extraction time; increasing ethanol concentration contributed to a higher CUPRAC value, while longer extraction time reduced this value. A similar pattern was observed for the influence of the investigated variables on FRAP. The influence of ethanol concentration was not significantly pronounced in the linear regression coefficients, but the quadratic coefficient indicated a maximum value at intermediate ethanol concentrations.

The negative quadratic terms for the solid-to-liquid ratio in both models (β_22_ = −109.51 and −110.96) were among the largest coefficients, indicating that solid-to-liquid ratio is a critical determinant of reducing-power responses: excessively high solvent volumes reduce the effective concentration of extracted phenolics in the final extract, directly diminishing the reducing equivalents measured per gram of extract, irrespective of total yield.

The phosphomolybdenum (PM) assay (R^2^ = 0.944) was primarily affected by ethanol concentration, with a positive linear regression coefficient (β_1_ = 0.351), followed by a somewhat weaker positive effect of the material-to-solvent ratio, while extraction time produced minimum values at intermediate levels. The highest PM value of 2.46 mmol TE/g was recorded for sample 14 (50% EtOH, 1:10 (*w*/*v*), 10 min).

Metal chelation activity exhibited the weakest model fit among all responses (R^2^ = 0.671), which, while statistically acceptable, reflects the inherent structural specificity of this mechanism. Metal chelation cannot be considered a highly predictive parameter, as the investigated variables did not show any significant influence on MC according to the regression coefficients, and it was therefore excluded from further multiobjective optimisation. Unlike radical-scavenging and reducing-power assays that respond proportionally to the total phenolic pool, metal chelation depends primarily on the presence of specific ortho-dihydroxy (catechol) and trihydroxy (galloyl) structural motifs, notably gallic acid, catechin, and protocatechuic acid subsequently identified in the optimised extract (Section 2.5), whose extraction efficiency does not scale linearly with overall phenolics yield across the experimental space. The residual variance not captured by the quadratic model likely reflects the non-linear contribution of minor chelating constituents whose concentrations vary independently of the dominant phenolic fractions. Despite the lower R^2^, the narrow absolute variation in metal chelation activity across all 15 BBD runs (15.62–20.22 mg EDTAE/g) suggests that the chelating capacity of *A. subcrenata* is robustly maintained across a broad range of extraction conditions [1,27].

Contextualized within the genus, the antioxidant activities of *A. subcrenata* are remarkably high. Authors of study [2] reported DPPH and ABTS activities of 191.2 and 372.5 mg TE/g for A. vulgaris methanol extracts, while comparable values have been documented for *A. mollis* [21,22,28] and *A. xanthochlora* [23]. The MAE-optimised *A. subcrenata* extract exhibited descriptively higher DPPH and ABTS values than those reported for A. vulgaris (432.05 vs. 191.2 mg TE/g and 1102.25 vs. 372.5 mg TE/g, respectively; Table 4); however, differences in the bases of expression across studies preclude a rigorous quantitative comparison. The remarkably high antioxidant activity observed in this study may reflect the inherent properties of this previously uninvestigated species; however, this enhancement could also be attributable to two synergistic factors: the superior extraction efficiency of MAE, which achieves rapid and uniform energy transfer that maximises cell disruption and phenolic release [7,8], and the high content of gallic acid, catechin, and quercetin hexosides subsequently identified in the optimised extract by LC-MS/MS analysis (Section 2.5). Gallic acid and catechin in particular are among the most potent hydrogen-atom donors in Folin-based and radical-scavenging assays, consistent with established structure–antioxidant activity relationships for phenolic acids and flavonoids [29,30,31], and their high abundance in the optimised *A. subcrenata* extract offers a plausible mechanistic explanation for the superior antioxidant profile observed here.

### 2.3. Enzyme Inhibitory Activity

The enzyme inhibitory activities of *A. subcrenata* extracts exhibited distinct response surface profiles for each target enzyme, confirming that the extraction of inhibitory bioactives is governed by compound-class-specific polarity and stability requirements (Table 2 and Table 5).

Cholinesterase inhibition (AChE, R^2^ = 0.941; BChE, R^2^ = 0.878) exhibited narrow absolute ranges of variation across all 15 BBD runs (AChE: 2.36–2.56 mg GALAE/g; BChE: 0.68–1.76 mg GALAE/g), suggesting that cholinesterase-inhibitory compounds in *A. subcrenata* are consistently recovered across a wide range of extraction conditions. AChE depends on ethanol concentration and extraction time, with a significant interaction coefficient (β_13_ = −0.052) between the investigated variables, indicating that their combined effect on the response is not simply additive. Ethanol concentration and extraction time influenced AChE through a significant quadratic term, pointing to a curved relationship with a minimum at extreme ethanol concentrations and a maximum at intermediate extraction times, while the linear terms were not significant. In contrast, BChE was primarily governed by the material-to-solvent ratio and extraction time, both showing significant quadratic effects, a minimum for the material-to-solvent ratio and a maximum for extraction time. The dominant BChE regression coefficient was the negative quadratic term for time (β_33_ = −0.425), indicating that both very short and very long extraction times are suboptimal, with prolonged extraction selectively degrading the primary BChE-inhibitory constituents, which may include quercetin-3-*O*-hexoside and flavonol glycosides identified in the optimised extract (Section 2.5) and previously shown to inhibit BChE in related species [2,32]. In comparison, AChE and BChE inhibitory activities of 2.11 and 1.20 mg GALAE/g have been reported for *A. vulgaris* methanol extracts [2], and comparable values have been documented for *A. xanthochlora* [25]. The MAE-optimised *A. subcrenata* extract (predicted AChE 2.48 mg GALAE/g; Table 4) is thus competitive with, or superior to, the best-characterised congeners within the genus, supporting its potential as a cholinesterase-inhibitory botanical ingredient relevant to neuroprotective applications [33].

Tyrosinase inhibition showed a broad variation across the BBD runs (50.28–62.38 mg KAE/g), with a well-fitted model (R^2^ = 0.942) dominated by significant positive linear effects of ethanol concentration (β_1_ = 2.225), solid-to-liquid ratio (β_2_ = 1.555), and negative linear effects of extraction time (β_3_ = −2.308). A significant negative quadratic coefficient for the solid-to-liquid ratio (β_22_ = −3.258) indicates a maximum in tyrosinase inhibition at moderate solid-to-liquid ratios. The response surface indicates that tyrosinase inhibition was maximised at high ethanol concentrations (≥ 60%) combined with moderate solid-to-liquid ratios (1:12.5–1:15) and short extraction times, consistent with the preferential extraction of moderately polar inhibitory compounds, likely including *p*-coumaric acid, caffeic acid, and chlorogenic acid subsequently identified by LC-MS/MS under optimal conditions (Section 2.5). These hydroxycinnamic acids are well-characterised inhibitors of tyrosinase that act through structural mimicry of the enzyme’s natural substrates, chelating the active-site copper ions and blocking catalytic oxidation, consistent with recent kinetic classifications of natural tyrosinase inhibitors acting via copper chelation [34,35,36]. The optimised extract (predicted 62.30 mg KAE/g; Table 4) appears higher than tyrosinase inhibitory values reported for *A. vulgaris* and *A. mollis* [23,24], suggesting that the higher content of hydroxycinnamic acid inhibitors in *A. subcrenata* may represent a species-specific advantage relevant to cosmeceutical applications targeting skin hyperpigmentation, warranting further investigation.

α-amylase inhibition was the most precisely predicted response in the entire BBD (R^2^ = 0.971), with experimental values ranging between 0.33 and 0.44 mmol ACAE/g. The positive linear effect of ethanol concentration (β_1_ = 0.043) indicates that α-amylase inhibition increased with increasing ethanol content, which is consistent with compounds that are likely to contribute to the observed inhibition, based on their documented α-amylase inhibitory activity and their abundance in the optimised extract (Section 2.5). As the assays were performed on the whole extract, the measured activity may also reflect additive or synergistic interactions among constituents rather than the action of individual compounds alone. Mechanistically, α-amylase inhibition by plant polyphenols proceeds via non-competitive inhibition, with quercetin, kaempferol, and chlorogenic acid forming non-covalent complexes at allosteric sites that reduce catalytic efficiency, a mechanism broadly supported by structure–activity analyses of flavonoid-mediated α-amylase inhibition [37,38,39]. The significant positive quadratic coefficient for the solid-to-liquid ratio (β_22_ = 0.015) indicates a minimum at intermediate ratios, meaning that both lower and higher solid-to-liquid ratios yielded greater α-amylase inhibition. The significant interaction between ethanol concentration and solid-to-liquid ratio suggests that the effect of one factor depends on the level of the other, underscoring the necessity of simultaneous optimisation. Extraction time had no significant influence on α-amylase inhibition. The predicted α-amylase inhibitory activity of the optimised extract (0.42 mmol ACAE/g; Table 4) is comparable to values reported for *A. vulgaris* and *A. xanthochlora* [2,25], confirming α-amylase inhibition as a consistent, genus-level property of *Alchemilla* rather than a species-specific trait.

Taken together, the enzyme inhibitory profile of *A. subcrenata* is distinguished within the genus principally by its tyrosinase inhibitory potency, attributable to the high concentration of hydroxycinnamic acid derivatives, and competitive AChE inhibition, potentially reflecting the combined contributions of quercetin-3-*O*-hexoside and catechin identified in the optimised extract (Section 2.5). These findings suggest that *A. subcrenata* warrants further investigation as a multi-target enzyme inhibitor with potential relevance to neuroprotective, antidiabetic, and cosmeceutical applications.

### 2.4. Optimisation and Model Validation

The obtained results emphasize the crucial role of extraction optimization, since both the concentrations of the identified compounds and the evaluated biological activities varied considerably under different extraction conditions. In some cases, nearly twofold differences were observed, demonstrating that extraction parameters markedly influence the chemical composition and biological potential of the extracts. The results of multiobjective optimisation using the desirability function approach identified the following optimal MAE conditions for *A. subcrenata*: ethanol concentration 60% (*v*/*v*), solid-to-liquid ratio 1:12.5 (*w*/*v*), and extraction time 10 min, with an overall desirability value of 0.80. TPC and TFC were excluded from multiobjective optimisation, as their values can directly influence antioxidant and enzyme activity, meaning not all responses are mutually independent. Thus, only mutually independent responses were included in the optimisation. Additionally, MC was excluded from optimisation due to low prediction of responses. These conditions represent a balanced compromise among the individual optima of the nine response variables, reflecting the competing polarity requirements of phenolic and flavonoid extraction and the thermal stability constraints of labile bioactive constituents. The relatively short extraction time (10 min) and intermediate ethanol concentration (60%) are consistent with the mechanistic findings presented earlier, where prolonged irradiation and excessively high or low ethanol concentrations were shown to compromise phenolic integrity and flavonoid recovery, respectively.

To validate model reliability, the extraction was performed in triplicate under the optimal conditions, and the resulting extract was subjected to the full panel of biological assays. The experimentally observed values were in close agreement with the model predictions across all responses (Table 4), with relative deviations ranging from only 1.3 to 2.9%. This close agreement supports the predictive capability of the fitted quadratic models and the reproducibility of the MAE process under the identified optimal conditions, indicating that RSM-guided optimisation successfully identified extraction conditions that maximise the simultaneous recovery of bioactive phenolics and biological activity from *A. subcrenata* [10,19]. It should be noted, however, that the lack-of-fit test was statistically significant (*p* ≤ 0.05) for eight of the twelve fitted models (TPC, TFC, DPPH, ABTS, CUPRAC, FRAP, MC, and BChE; Supplementary Table S1).

This is likely attributable to the very low pure-error variance typical of Box–Behnken designs with a limited number of centre-point replicates: in the present dataset, pure error accounted for well under 1% of the total sum of squares for all affected responses, ranging from approximately 0.00001% (ABTS) to 0.53% (MC) (Supplementary Table S1), which is sufficient to inflate the lack-of-fit F-ratio even when the quadratic model adequately describes the underlying response surface. Given the high R^2^ values obtained (0.878–0.971) and the close experimental validation described above, the models were nonetheless considered fit for the purpose of process optimisation.

Additional model diagnostics, including observed-versus-predicted and residual-versus-predicted plots for all responses (Supplementary Figures S2 and S3), showed no systematic trend or funnel pattern in the residuals across the predicted range, further supporting the adequacy of the fitted models for process optimisation purposes. To the best of our knowledge, this is the first application of MAE combined with a Box–Behnken design to any *Alchemilla* species, precluding direct comparison with MAE-optimised extraction data for congeneric taxa. Despite these advantages, the practical limitations of MAE should be acknowledged. Microwave heating can generate localised temperature gradients and hot spots that, particularly at prolonged irradiation, may promote thermal degradation of thermolabile phenolics [40], an effect consistent with the decline in several responses observed at the longest extraction times in the present study. As the system was operated at fixed microwave power without continuous internal temperature monitoring, the bulk temperature was not recorded throughout each run, and minor solvent evaporation cannot be fully excluded despite the use of a reflux condenser. Extraction efficiency is also matrix-dependent, so the optimal conditions identified here are specific to the leaves of *A. subcrenata* and may require re-optimisation for other plant parts [41]. Finally, although the short extraction time and moderate solvent demand are favourable for scale-up, the transition from laboratory-scale modified equipment to dedicated industrial MAE systems introduces additional engineering considerations, including uniform field distribution and heat management, that remain to be addressed.

### 2.5. LC-MS/MS Phenolic Profile

The *A. subcrenata* extract obtained by MAE at RSM-optimised conditions (ethanol concentration 60% (*v*/*v*), solid-to-liquid ratio 1:12.5 (*w*/*v*)) was analysed using LC-MS/MS. The analysis was consistent with standard practice in extraction optimisation studies where comprehensive phytochemical characterisation is reserved for the optimised extract [9]. The optimised *A. subcrenata* extract revealed a structurally diverse and quantitatively rich phenolic profile comprising 28 individual compounds across six chemical classes (Table 6), including the alicyclic acid quinic acid, co-quantified by the same method.

Analysed but not detected: Scopoletin, 3,4-Dimethoxycinnamic acid, daidzein, Epicatechin, Myricetin, cinnamic acid, umbelliferone, genistein, baicalein, Matairesinol, Secoisolariciresinol, baicalin, Epigallocatechin gallate, Amentoflavone, apiin, o-coumaric acid.

The combined content of the 28 quantified phenolic compounds amounted to 1599.7 μg/g extract, with flavonoid glycosides and aglycones collectively constituting the dominant fraction. Among all identified compounds, quercetin-3-*O*-hexoside was the single most abundant constituent (666.5 μg/g), consistent with the well-established predominance of quercetin hexosides in *Alchemilla* species [1,2,42]. Its high concentration in the optimised extract underscores the capacity of intermediate ethanol concentrations (60%) and brief microwave irradiation to maximally recover polar flavonoid glycosides while limiting their thermal degradation. The second most abundant compound was gallic acid (196.4 μg/g), a ubiquitous hydroxybenzoic acid in Rosaceae species frequently associated with pronounced antioxidant and enzyme inhibitory activities [24,25,43]. Catechin (161.1 μg/g) and quinic acid (152.4 μg/g) were also present in high concentrations, reflecting the characteristic tannin-related chemistry of *Alchemilla* and the broader Rosaceae family [1].

The flavonol glycoside fraction was further represented by kaempferol-3-*O*-glucoside (103.3 μg/g), rutin (35.61 μg/g), and quercitrin (11.61 μg/g), alongside the free aglycones quercetin (11.50 μg/g), isorhamnetin (2.09 μg/g), and kaempferol (1.74 μg/g). The co-occurrence of both glycosylated and free forms of quercetin and kaempferol derivatives suggests that the optimised MAE conditions facilitated efficient extraction of intact glycosides while the brief microwave irradiation limited hydrolytic degradation to aglycones to a minor extent, consistent with findings for MAE of flavonoid-rich plant matrices [7]. Among hydroxycinnamic acid derivatives, *p*-coumaric acid (50.68 μg/g) and chlorogenic acid (45.32 μg/g) were the most prominent, followed by caffeic acid (23.13 μg/g) and ferulic acid (20.93 μg/g). The preferential recovery of these compounds at 60% ethanol and 10 min extraction time is consistent with the tyrosinase and α-amylase inhibitory profiles, given that hydroxycinnamic acids are well-established multi-target enzyme inhibitors acting through copper chelation and allosteric binding mechanisms [34,35,44].

Among the flavone subclass, luteolin-7-*O*-glucoside (23.56 μg/g) and apigenin-7-*O*-glucoside (4.77 μg/g) were detected alongside their respective aglycones (luteolin, 3.55 μg/g; apigenin, 1.40 μg/g) and chrysoeriol (2.63 μg/g). The flavone C-glycoside vitexin was detected at trace levels (0.051 μg/g), consistent with the low abundance of C-glycosylated flavones typically reported in Rosaceae species [1]. The presence of naringenin (0.353 μg/g) and aesculetin (6.697 μg/g) is noteworthy, as these compound classes have not been previously reported in *A. subcrenata*, representing novel additions to the known phytochemical inventory of this species.

Collectively, the phenolic profile of *A. subcrenata* is consistent with the phenolic fingerprint characteristic of the genus [1,2], while also revealing quantitative distinctions relative to better-characterised congeners. Notably, the combined content of the quantified phenolic compounds (1599.7 μg/g) and the dominance of quercetin-3-O-hexoside are broadly consistent with the phenolic fingerprint reported for other *Alchemilla* species, although direct quantitative comparison is limited by differences in extraction solvents and methods across studies [2,23,24]. This profile reflects both the efficiency of the optimised MAE protocol and the inherent phytochemical richness of *A. subcrenata*. The co-occurrence of gallic acid, catechin, quercetin hexosides, and hydroxycinnamic acids with complementary antioxidant and enzyme inhibitory mechanisms provides a mechanistic basis for the multi-target bioactivity profile, and underscores the promise of *A. subcrenata* as a source of functional phytochemicals warranting further investigation for potential nutraceutical, pharmaceutical, and cosmeceutical applications.

## 3. Materials and Methods

### 3.1. Plant Material

The leaves of *A. subcrenata* Buser were collected during the flowering stage at Vlasina Lake, Republic of Serbia, in August 2023, and were kindly provided by Mint Pharm company (Bačka Palanka, Serbia). The specimen voucher (*A. subcrenata* Buser, 2-0016) was prepared and identified by Milica Rat, Ph.D., and deposited at the Herbarium of the Department of Biology and Ecology (BUNS Herbarium), University of Novi Sad, Faculty of Sciences, Republic of Serbia. After collection, the plant material was air-dried at room temperature in a well-ventilated and shaded area, then ground into a fine powder using a laboratory mill and stored in airtight containers until extraction.

### 3.2. Chemicals and Reagents

Folin–Ciocalteu reagent, sodium carbonate, aluminum trichloride, gallic acid, rutin, 2,2-diphenyl-1-picrylhydrazyl (DPPH), 2,2′-azino-bis(3-ethylbenzothiazoline-6-sulfonic acid) (ABTS), 2,4,6-tris(2-pyridyl)-s-triazine (TPTZ), copper(II) chloride, neocuproine, ammonium molybdate, acetylcholinesterase (AChE, EC 3.1.1.7, from electric eel), butyrylcholinesterase (BChE, EC 3.1.1.8, from equine serum), tyrosinase (EC 1.14.18.1, from mushroom), α-amylase (EC 3.2.1.1, from porcine pancreas), acetylthiocholine iodide, butyrylthiocholine iodide, 5,5′-dithiobis(2-nitrobenzoic acid) (DTNB), L-DOPA, starch, galantamine, kojic acid, acarbose, and all individual phenolic standards used for LC-MS/MS quantification were purchased from Sigma-Aldrich (St. Louis, MO, USA) and/or Extrasynthese (Genay, France)**.** Ethanol of analytical grade and HPLC-grade acetonitrile were used as extraction and mobile phase solvents, respectively. All other reagents and solvents were of analytical grade. Ultrapure water was used throughout all analyses.

### 3.3. Microwave-Assisted Extraction and Experimental Design

Microwave-assisted extraction (MAE) was performed using a microwave oven (ME71A, Samsung, Port Klang, Malaysia) modified for laboratory use, equipped with a condenser to minimize solvent evaporation. Extraction was carried out in a 500 mL round-bottom flask. Microwave power was kept constant at 600 W throughout all experiments. In each run, 5 g of dried leaves was extracted with the corresponding volume of aqueous ethanol according to the assigned solid-to-liquid ratio (50, 75, and 100 mL for ratios of 1:10, 1:15, and 1:20 (*w*/*v*), respectively), and the mixture was subjected to microwave irradiation for the designated extraction time. After extraction, the mixtures were filtered through qualitative filter paper (pore size 4–12 μm; Schleicher & Schuell, Dassel, Germany), and the resulting crude extracts were stored at 4 °C until analysis. Because the modified microwave system operated at atmospheric pressure and was fitted with a reflux condenser, the extraction mixture was maintained at the boiling point of the aqueous-ethanol solvent (approximately 98 °C); evaporated solvent was continuously condensed and returned to the flask, so that the temperature was inherently limited by the solvent boiling point rather than by active regulation. The extraction yield under the optimised conditions was determined gravimetrically by concentrating the extract under reduced pressure on a rotary evaporator and drying it to constant mass, and amounted to approximately 20% (*w*/*w*) relative to the dry plant material. The extraction conditions were optimized using Response Surface Methodology (RSM) based on a Box–Behnken design (BBD). Three independent variables were investigated: ethanol concentration (X_1_: 30–70%, *v*/*v*), solid-to-liquid ratio (X_2_: 1:10–1:20, *w*/*v*), and extraction time (X_3_: 10–30 min). Each variable was studied at three coded levels: low (−1), medium (0), and high (+1), as detailed in Table 7. The BBD consisted of 15 experimental runs, including three replicates at the central point to estimate pure experimental error. The experimental responses were total phenolic content (TPC), total flavonoid content (TFC), antioxidant activities (DPPH, ABTS, CUPRAC, FRAP), metal chelating activity (MC), phosphomolybdenum total antioxidant capacity (PM), and enzyme inhibitory activities (AChE, BChE, tyrosinase, and α-amylase).

The experimental data were fitted to the following second-order polynomial model:

Y = β_0_ + β_1_X_1_ + β_2_X_2_ + β_3_X_3_ + β_11_X_1_^2^ + β_22_X_2_^2^ + β_33_X_3_^2^ + β_12_X_1_X_2_ + β_13_X_1_X_3_ + β_23_X_2_X_3_

where Y is the predicted response, β_0_ is the intercept, β_1_, β_2_, β_3_ are the linear coefficients, β_11_, β_22_, β_33_ are the quadratic coefficients, and β_12_, β_13_, β_23_ are the interaction coefficients. The MAE aimed to prepare extracts with the highest levels of all investigated parameters. As TPC and TFC were output variables that directly influenced other responses, and MC could not be considered a highly predictive parameter (R^2^ = 0.671), the multiobjective optimisation included maximising antioxidant and enzyme inhibitory activities (DPPH, ABTS, CUPRAC, FRAP, PM, AChE, BChE, tyrosinase, and α-amylase). Optimal extraction conditions were verified experimentally in three independent replicate runs.

Multi-response optimisation was performed using the desirability function approach of Derringer and Suich [45], whereby each individual response was transformed into a dimensionless desirability score (0–1) and combined into an overall composite desirability (D), which was maximised to identify the optimal extraction conditions.

### 3.4. Determination of Total Phenolic and Flavonoid Contents

Total phenolic content (TPC) was determined using the Folin–Ciocalteu colorimetric method with minor modifications, as described previously [46]. Briefly, 25 µL of extract was mixed with 125 µL of diluted Folin–Ciocalteu reagent (1:10, *v*/*v*) in a 96-well microplate, followed by the addition of 100 µL of sodium carbonate solution (75 g/L). After incubation for 2 h at room temperature in the dark, absorbance was measured at 760 nm using a microplate reader. Gallic acid was used for calibration (0.025–0.500 mg/mL), and results were expressed as milligrams of gallic acid equivalents per gram of extract (mg GAE/g extract).

Total flavonoid content (TFC) was determined by the aluminum chloride colorimetric assay according to the procedure of Grochowski et al. [24]. Briefly, 25 µL of extract was mixed with 25 µL of aluminum trichloride solution (2%, *w*/*v* in methanol) and 200 µL of methanol in a 96-well microplate, while a corresponding blank was prepared by replacing aluminum trichloride with methanol. After incubation for 1 h at room temperature, absorbance was measured at 415 nm. Rutin was used as the reference standard (0.025–0.500 mg/mL), and results were expressed as milligrams of rutin equivalents per gram of extract (mg RE/g extract).

### 3.5. LC-MS/MS Analysis of Phenolic Compounds

The content of 44 phenolic compounds in the optimized extract was analyzed by liquid chromatography coupled with tandem mass spectrometry (LC-MS/MS), following the method of Orčić et al. [26], with minor modifications. Briefly, the mobile phase flow rate was adjusted to 0.5 mL min^−1^, and hyperoside and quercetin-3-O-glucoside were quantified together as quercetin-3-O-hexoside. Prior to LC-MS/MS analysis, the extract was evaporated to dryness and subsequently reconstituted in the appropriate solvent to final concentrations of 0.2, 2, and 20 mg mL^−1^ to ensure that the concentrations of all analytes fell within the corresponding linear calibration ranges. Analyses were performed on an Agilent 1200 Series HPLC system coupled to an Agilent 6410A Triple Quad mass spectrometer equipped with an electrospray ionization (ESI) source (Agilent Technologies, Santa Clara, CA, USA). Separation was performed on a Zorbax Eclipse XDB-C18 column (50 mm × 4.6 mm, 1.8 µm) held at 50 °C, using mobile phase A (0.05% aqueous formic acid) and B (methanol) at a flow rate of 0.5 mL/min in gradient mode (0 min, 30% B; 6 min, 70% B; 18 min, 100% B; 24 min, 100% B; 6 min re-equilibration). The injection volume was 5 µL. Detection was performed in negative ionization mode with the following source parameters: nebulizer gas (N_2_) pressure 40 psi, drying gas (N_2_) flow 9 L/min at 350 °C, and capillary voltage 4 kV. Data acquisition was performed in dynamic Multiple Reaction Monitoring (dMRM) mode using compound-specific precursor/product ion transitions, fragmentor voltages, and collision energies. Quantification was based on external calibration curves constructed from authentic standards. Peak areas were determined and processed using Agilent MassHunter Workstation Software, and the concentrations of the analyzed compounds were calculated using OriginPro software (version 8.0; OriginLab Corporation, Northampton, MA, USA). The retention times and MS parameters are given in Table S2 in the Supplementary Materials. Results were expressed as micrograms per gram of dry extract (µg/g extract).

### 3.6. Antioxidant and Enzyme Inhibitory Assays

All antioxidant and enzyme inhibitory assays were performed in 96-well microplates, following the procedures of Uysal et al. [25] and Grochowski et al. [24]; the modifications consisted of adapting the assays to the 96-well microplate format, with extracts dissolved at 1 mg/mL before analysis. All measurements were performed in triplicate, and appropriate blanks and controls were included in each assay.

Antioxidant assays. For the DPPH radical scavenging assay, the sample solution was added to a 0.004% (*w*/*v*) methanolic DPPH solution, and the absorbance was read at 517 nm after 30 min of incubation at room temperature in the dark. For the ABTS assay, the ABTS•^+^ radical cation was generated by reacting a 7 mM ABTS solution with 2.45 mM potassium persulfate and allowing the mixture to stand in the dark at room temperature for 12–16 h; the working solution was then diluted with methanol to an absorbance of 0.70 ± 0.02 at 734 nm, and the sample absorbance was measured at 734 nm after 30 min. Both radical-scavenging results were expressed as mg Trolox equivalents per gram of extract (mg TE/g extract).

For the CUPRAC assay, the sample solution was added to a freshly prepared reaction mixture containing CuCl_2_ (10 mM), neocuproine (7.5 mM), and ammonium acetate buffer (1 M, pH 7.0); the absorbance was read at 450 nm after 30 min at room temperature. For the FRAP assay, the sample solution was added to FRAP reagent consisting of acetate buffer (0.3 M, pH 3.6), TPTZ (10 mM in 40 mM HCl), and FeCl_3_ (20 mM) in a 10:1:1 (*v*/*v*/*v*) ratio; the absorbance was read at 593 nm after 30 min at room temperature. CUPRAC and FRAP results were expressed as mg TE/g extract. Together, the DPPH and ABTS assays represent hydrogen-atom/electron-transfer-based radical-scavenging methods, whereas CUPRAC and FRAP are electron-transfer-based reducing-power assays, providing complementary mechanistic insight into the antioxidant behaviour of the extracts [47,48].

For the phosphomolybdenum assay, the sample solution was combined with reagent solution (0.6 M sulfuric acid, 28 mM sodium phosphate, and 4 mM ammonium molybdate) and incubated at 95 °C for 90 min, after which the absorbance was measured at 695 nm; results were expressed as mmol TE/g extract. For metal chelating activity, the sample solution was added to FeCl_2_ (2 mM), the reaction was initiated with ferrozine (5 mM), and after 10 min at room temperature, the absorbance was read at 562 nm against a ferrozine-free blank; results were expressed as mg EDTA equivalents per gram of extract (mg EDTAE/g extract).

Enzyme inhibitory assays. Cholinesterase inhibition was determined by Ellman’s method [49]: the sample solution (50 µL) was mixed with DTNB (125 µL) and AChE or BChE solution (25 µL) in Tris–HCl buffer (pH 8.0) and incubated at 25 °C for 15 min, after which the reaction was initiated by adding the substrate (25 µL of acetylthiocholine iodide for AChE or butyrylthiocholine iodide for BChE). The absorbance was measured at 405 nm, and results were expressed as mg galantamine equivalents per gram of extract (mg GALAE/g extract). Tyrosinase inhibition was measured by the dopachrome method using L-DOPA as substrate: the sample solution (25 µL) was combined with tyrosinase solution (40 µL) and phosphate buffer (100 µL, pH 6.8) and incubated at 25 °C for 15 min, after which the reaction was initiated by adding L-DOPA (40 µL); the absorbance was read at 492 nm and results were expressed as mg kojic acid equivalents per gram of extract (mg KAE/g extract). α-Amylase inhibition was determined by the iodine–potassium iodide method [50]: the sample solution was mixed with α-amylase solution in phosphate buffer (pH 6.9 with 6 mM NaCl) and pre-incubated at 37 °C for 10 min, after which starch solution was added and incubated at 37 °C for 10 min; the reaction was stopped with HCl, iodine–potassium iodide solution was added, and the absorbance was read at 630 nm. Results were expressed as mmol acarbose equivalents per gram of extract (mmol ACAE/g extract).

### 3.7. Statistical Analysis

All assays were performed in triplicate (*n* = 3) and results are expressed as mean ± standard deviation. The relationship between the three independent variables and each response was modelled by second-order polynomial regression within the Box–Behnken design. Model adequacy and the significance of individual regression terms were evaluated by analysis of variance (ANOVA), including the coefficient of determination (R^2^) and adjusted R^2^, with *p* ≤ 0.05 considered statistically significant (Table 2). The full ANOVA, including the lack-of-fit test, for all twelve responses is provided in Supplementary Table S1, and the desirability profiles used for multi-response optimisation are shown in Supplementary Figure S1. Observed-versus-predicted and predicted-versus-residual plots for all twelve responses are provided in Supplementary Figures S2 and S3, respectively. All statistical analyses were performed using STATISTICA 14 (StatSoft Inc., Tulsa, OK, USA).

## 4. Conclusions

This study systematically optimised microwave-assisted extraction conditions for *A. subcrenata* Buser using a Box–Behnken experimental design within a Response Surface Methodology framework. Second-order polynomial models showed statistically adequate predictive power across eleven of twelve response variables (R^2^ = 0.878–0.971), and the multiobjective optimisation identified optimal extraction conditions of 60% ethanol, 1:12.5 (*w*/*v*) solid-to-liquid ratio, and 10 min extraction time, with excellent agreement between predicted and experimentally validated values confirming the reliability and reproducibility of the fitted models. Under these optimised conditions, *A. subcrenata* exhibited exceptional antioxidant activities across all six assays and significant inhibitory potency against all four target enzymes, with values appearing higher than those reported for the best-characterised congeners of the genus obtained by conventional extraction methods, including *A. vulgaris* and *A. mollis*. LC-MS/MS characterisation of the optimised extract identified 28 phenolic compounds across six chemical classes, dominated by quercetin-3-O-hexoside, gallic acid, catechin, quinic acid, and kaempferol glycosides. The presence of naringenin and aesculetin represents a novel phytochemical finding for this species. The rich content of quercetin hexosides and hydroxycinnamic acids provides a direct mechanistic basis for the observed multi-target bioactivity profile, linking extract composition to antioxidant, tyrosinase inhibitory, and α-amylase inhibitory activities. These findings establish *A. subcrenata* as an exceptionally rich and previously underexplored source of bioactive phenolics, highlighting both the efficiency of MAE as a green extraction strategy and the species-specific phytochemical potential of this neglected *Alchemilla* taxon. From a practical standpoint, the identified optimal conditions, 60% ethanol, 1:12.5 (*w*/*v*) solid-to-liquid ratio, and 10 min extraction time, are operationally straightforward and compatible with scale-up, positioning *A. subcrenata* as a promising candidate warranting further investigation for potential nutraceutical, pharmaceutical, and cosmeceutical applications. The present study is limited to in vitro assessments of bioactivity; the lack of in vivo validation and bioavailability data represents the primary limitation of the present work. Future research should address the bioavailability and in vivo efficacy of key phenolic constituents, particularly quercetin-3-*O*-hexoside, gallic acid, and hydroxycinnamic acid derivatives. Further work should also focus on isolation and characterisation of individual bioactive compounds, scaling up the optimised MAE process for industrial applications, and extending the phytochemical characterisation to include ellagitannins and other high-molecular-weight phenols not captured by the current LC-MS/MS method.

## Figures and Tables

**Table 1:** Total phenolic and flavonoid contents ^1^ in the examined extracts.

Samples	Factor Levels			Responses	
	% EtOH (X1)	M/S Ratio (X2)	t (min) (X3)	Total Phenolics (mg GAE/g) ^2^	Total Flavonoids (mg RE/g) ^3^
1	50	1:20	30	65.35 ± 0.40 ^c^	32.70 ± 0.52 ^d^
2	30	1:15	10	64.82 ± 0.03 ^c^	40.68 ± 0.94 ^a^
3	50	1:15	20	71.52 ± 0.70 ^a^	37.12 ± 0.31 ^b^
4	50	1:10	30	66.26 ± 0.14 ^b,c^	34.84 ± 0.37 ^c^
5	30	1:20	20	48.84 ± 0.73 ^f^	26.08 ± 0.31 ^g^
6	70	1:20	20	51.69 ± 0.76 ^e^	22.32 ± 0.71 ^h^
7	50	1:20	10	67.67 ± 0.47 ^b^	35.59 ± 0.18 ^c^
8	50	1:15	20	72.71 ± 0.41 ^a^	37.82 ± 0.30 ^b^
9	70	1:10	20	44.10 ± 0.16 ^g^	19.54 ± 0.15 ^i^
10	30	1:15	30	49.51 ± 0.66 ^f^	25.14 ± 0.19 ^g^
11	30	1:10	20	54.50 ± 0.83 ^d^	30.88 ± 0.17 ^f^
12	50	1:15	20	71.05 ± 0.24 ^a^	37.21 ± 0.12 ^b^
13	70	1:15	30	71.87 ± 0.37 ^a^	38.04 ± 0.36 ^b^
14	50	1:10	10	72.71 ± 0.70 ^a^	38.20 ± 0.14 ^b^
15	70	1:15	10	71.30 ± 0.63 ^a^	32.83 ± 0.18 ^d^

^1^ values are means ± SD of three measurements. Means within each row with different letters (a–i) differ significantly (*p* ≤ 0.05). ^2^ mg Gallic acid equivalents per gram of extract. ^3^ mg Rutin equivalents per gram of extract.

**Table 2:** Regression coefficients (β) and determination coefficients (R^2^) for the quadratic models of MAE responses of *A. subcrenata*.

Parm	TPC	TFC	DPPH	ABTS	CUPRAC	FRAP	MC	PM	AChE	BChE	Tyrosinase	α-Amylase
***β*** **_0_**	71.7603	37.38491	338.958	921.870	592.019	461.642	20.06960	1.955818	2.495362	1.391782	59.85163	0.380904
***β_1_***	NS	NS	58.629	218.323	65.098	NS	NS	0.350611	NS	NS	2.22477	0.042594
***β*** **_11_**	−12.8010	−6.91897	−147.019	−554.749	NS	−59.226	NS	NS	0.042008	NS	NS	NS
***β*** **_2_**	NS	NS	NS	NS	NS	NS	NS	0.150374	NS	NS	1.55512	NS
***β*** **_22_**	−9.1776	−5.76110	−89.476	−464.453	−109.511	−110.958	NS	NS	NS	0.260893	−3.25771	0.015044
***β*** **_3_**	NS	NS	NS	NS	−56.948	−44.859	NS	NS	NS	NS	−2.30777	NS
***β*** **_33_**	NS	NS	82.852	367.494	164.333	119.525	NS	0.201637	−0.066548	−0.424834	NS	NS
***β*** **_12_**	NS	NS	NS	NS	NS	NS	NS	NS	NS	NS	NS	0.014768
***β*** **_13_**	NS	5.18563	NS	NS	NS	NS	NS	NS	−0.052079	−0.166015	NS	NS
***β*** **_23_**	NS	NS	NS	NS	NS	NS	NS	NS	NS	NS	NS	NS
**R^2^**	**0.87687**	**0.90789**	**0.92331**	**0.95284**	**0.94217**	**0.96179**	**0.67064**	**0.94438**	**0.94137**	**0.8782**	**0.94237**	**0.97071**
**Adj R^2^**	**0.65498**	**0.74208**	**0.78526**	**0.86794**	**0.83807**	**0.89302**	**0.0778**	**0.84427**	**0.83584**	**0.65896**	**0.83865**	**0.91800**

Values shown in red denote statistically significant regression terms (*p* ≤ 0.05). NS—non-significant.

**Table 3:** Antioxidant properties ^1^ of the tested extracts.

No	Factor Levels			Responses					
	% EtOH (X1)	M/S ratio (X2)	t (min) (X3)	DPPH (mg TE/g) ^2^	ABTS (mg TE/g) ^2^	CUPRAC (mg TE/g) ^2^	FRAP (mg TE/g) ^2^	MC (mg EDTAE/g) ^3^	PM (mmol TE/g) ^4^
**1**	50	1:20	30	367.62 ± 11.91 ^a,b^	941.60 ± 1.68 ^b,c,d^	591.43 ± 9.20 ^c^	450.38 ± 6.28 ^c^	19.40 ± 0.21 ^a,b^	1.87 ± 0.07 ^b,c^
**2**	30	1:15	10	259.27 ± 19.66 ^d^	734.99 ± 22.05 ^e^	694.39 ± 12.62 ^b^	555.09 ± 5.94 ^a,b^	19.68 ± 0.22 ^a,b^	1.74 ± 0.02 ^c,d^
**3**	50	1:15	20	230.23 ± 10.44 ^d^	922.05 ± 26.70 ^c,d^	589.73 ± 5.71 ^c^	460.55 ± 27.78 c	19.77 ± 0.44 ^a,b^	1.90 ± 0.03 ^b,c^
**4**	50	1:10	30	242.16 ± 9.25 ^d^	775.89 ± 18.77 ^e^	568.22 ± 3.23 ^c^	405.87 ± 23.87 ^d^	20.21 ± 0.29 ^a^	1.95 ± 0.12 ^b^
**5**	30	1:20	20	59.92 ± 8.15 ^g^	328.31 ± 9.94 ^h^	357.52 ± 7.64 ^f^	279.70 ± 8.53 ^e^	19.61 ± 0.25 ^a,b^	1.21 ± 0.02 ^e^
**6**	70	1:20	20	178.35 ± 6.88 ^e^	441.69 ± 18.69 ^g^	463.93 ± 37.30 ^d^	311.46 ± 13.94 ^e^	17.24 ± 0.18 ^e^	1.82 ± 0.03 ^b,c^
**7**	50	1:20	10	314.25 ± 5.99 ^c^	766.23 ± 17.24 ^e^	652.25 ± 6.88 ^b^	477.74 ± 8.93 ^c^	18.64 ± 0.07 ^c,d^	1.82 ± 0.05 ^b,c^
**8**	50	1:15	20	340.61 ± 15.08 ^b,c^	921.90 ± 18.14 ^c,d^	593.27 ± 3.39 ^c^	460.80 ± 7.53 ^c^	20.22 ± 0.55 ^a^	1.97 ± 0.02 ^b^
**9**	70	1:10	20	54.68 ± 9.99 ^g^	461.97 ± 22.35 ^g^	414.26 ± 11.97 ^e^	279.93 ± 9.62 ^e^	15.62 ± 0.23 ^f^	1.93 ± 0.01 ^b^
**10**	30	1:15	30	83.90 ± 9.85 ^f,g^	563.97 ± 27.61 ^f^	471.10 ± 9.57 ^d^	370.76 ± 3.86 ^d^	17.70 ± 0.38 ^d,e^	1.28 ± 0.04 ^e^
**11**	30	1:10	20	116.90 ± 5.06 ^f^	417.11 ± 37.32 ^g^	492.09 ± 5.88 ^d,e^	294.74 ± 4.68 ^e^	17.35 ± 0.06 ^e^	1.57 ± 0.03 ^d^
**12**	50	1:15	20	340.25 ± 21.56 ^b,c^	892.34 ± 13.40 ^d^	593.06 ± 17.73 ^c^	463.58 ± 4.69 ^c^	20.22 ± 0.29 ^a^	1.99 ± 0.05 ^b^
**13**	70	1:15	30	364.52 ± 22.63 ^a,b^	1040.91 ± 22.41 ^a^	815.82 ± 40.26 ^a^	577.85 ± 2.14 ^a,b^	19.24 ± 0.22 ^b,c^	2.42 ± 0.10 ^a^
**14**	50	1:10	10	405.31 ± 30.11 ^a^	1009.83 ± 33.55 ^a,b^	775.46 ± 9.59 ^a^	546.84 ± 7.28 ^b^	19.71 ± 0.49 ^a,b^	2.46 ± 0.09 ^a^
**15**	70	1:15	10	391.47 ± 15.46 ^a^	973.11 ± 45.57 ^a,b,c^	780.04 ± 11.31 ^a^	583.16 ± 13.87 ^a^	18.96 ± 0.18 ^b,c^	2.43 ± 0.07 ^a^

^1^ values are means ± SD of three measurements. Means within each row with different letters (a–h) differ significantly (*p* ≤ 0.05). ^2^ mg Trolox equivalents per g of extract. ^3^ mg EDTAE per g of extract. ^4^ mmol Trolox equivalents per g of extract.

**Table 4:** Predicted and observed values of each individual response at optimal MAE conditions for *A. subcrenata* Buser extract (ethanol concentration 60% (*v*/*v)*, solid-to-liquid ratio 1:12.5 (*w*/*v*), extraction time 10 min).

Optimal Conditions	Investigated Response ^a^	
	Predicted	Observed ^b^
DPPH (mg TE/g)	421.92	432.05 ± 8.64
ABTS (mg TE/g)	1081.70	1102.25 ± 22.05
CUPRAC (mg TE/g)	787.93	801.33 ± 16.03
FRAP (mg TE/g)	588.49	600.85 ± 12.02
PM (mmol TE/g)	2.438	2.492 ± 0.05
AChE (mg GALAE/g)	2.475	2.507 ± 0.04
BChE (mg GALAE/g)	0.965	0.993 ± 0.02
Tyrosinase (mg KAE/g)	62.298	63.357 ± 1.12
α-amylase (mmol ACAE/g)	0.422	0.430 ± 0.008
Composite desirability	0.80061	

Optimal conditions: ethanol concentration 60% (*v*/*v*), solid-to-liquid ratio 1:12.5 (*w*/*v*), extraction time 10 min. ^a^ Metal chelation activity was excluded from the validation table due to the lowest model fit among all responses (R^2^ = 0.671); predicted and observed values fell within the range documented across all 15 BBD runs (15.62–20.22 mg EDTAE/g), confirming adequate model performance for this response. ^b^ Mean ± SD; *n* = 3 independent extraction replicates performed under optimal conditions. Relative deviations between predicted and observed values: 1.3–2.9%.

**Table 5:** Enzyme inhibitory properties ^1^ of the tested extracts.

Samples	Factor Levels			Response			
	% EtOH (X1)	Ratio M/S Ratio (X2)	t (min) (X3)	AChE (mg GALAE/g) ^2^	BChE (mg GALAE/g) ^2^	Tyrosinase (mg KAE/g) ^3^	α-Amylase (mmol ACAE/g) ^4^
**1**	50	1:20	30	2.38 ± 0.04 ^e,f^	1.51 ± 0.12 ^a,b^	51.64 ± 0.13 ^e,f^	0.39 ± 0.01 ^c,d,e,f^
**2**	30	1:15	10	2.41 ± 0.02 ^d,e,f^	1.10 ± 0.37 ^b,c,d,e^	54.22 ± 1.32 ^d^	0.36 ± 0.01 ^f,g,h^
**3**	50	1:15	20	2.48 ± 0.01 ^a,b,c,d^	1.39 ± 0.14 ^a,b,c^	53.98 ± 0.21 ^d^	0.43 ± 0.02 ^a,b,c^
**4**	50	1:10	30	2.36 ± 0.03 ^f^	1.31 ± 0.18 ^a,b,c,d^	52.92 ± 1.57 ^d,e^	0.42 ± 0.01 ^a,b,c^
**5**	30	1:20	20	2.56 ± 0.03 ^a^	1.76 ± 0.04 ^a^	50.28 ± 0.33 ^f^	0.37 ± 0.01 ^e,f,g^
**6**	70	1:20	20	2.51 ± 0.02 ^a,b,c^	1.76 ± 0.03 ^a^	56.18 ± 0.12 ^c^	0.42 ± 0.02 ^a,b,c^
**7**	50	1:20	10	2.48 ± 0.04 ^a,b,c,d^	1.12 ± 0.08 ^b,c,d^	56.02 ± 0.49 ^c^	0.41 ± 0.01 ^b,c,d,e^
**8**	50	1:15	20	2.52 ± 0.02 ^a,b^	1.40 ± 0.05 ^a,b^	60.15 ± 0.51 ^b^	0.38 ± 0.01 ^d,e,f^
**9**	70	1:10	20	2.53 ± 0.09 ^a,b^	1.76 ± 0.16 ^a^	56.33 ± 0.36 ^c^	0.44 ± 0.01 ^a,b^
**10**	30	1:15	30	2.50 ± 0.09 ^a,b,c^	1.48 ± 0.10 ^a,b^	53.57 ± 0.28 ^d^	0.33 ± 0.01 ^h^
**11**	30	1:10	20	2.47 ± 0.05 ^bc,de^	1.67 ± 0.17 ^a^	56.17 ± 1.37 ^c^	0.34 ± 0.01 ^g,h^
**12**	50	1:15	20	2.49 ± 0.02 ^a,b,c,d^	1.38 ± 0.11 ^a,b,c,d^	59.19 ± 0.49 ^b^	0.41 ± 0.01 ^b,c,d^
**13**	70	1:15	30	2.42 ± 0.07 ^c,d,e,f^	0.68 ± 0.07 ^e^	57.16 ± 0.43 ^c^	0.44 ± 0.01 ^a^
**14**	50	1:10	10	2.40 ± 0.06 ^d,e,f^	0.97 ± 0.14 ^c,d,e^	61.14 ± 0.23 ^a,b^	0.42 ± 0.02 ^a,b,c^
**15**	70	1:15	10	2.54 ± 0.04 ^a,b^	0.96 ± 0.08 ^d,e^	62.38 ± 0.48 ^a^	0.44 ± 0.01 ^a,b^

^1^ values are means ± SD of three measurements. Means within each row with different letters (a–h) differ significantly (*p* ≤ 0.05). ^2^ mg Galantamine equivalents per g of extract. ^3^ mg Kojic acid per g of extract. ^4^ mmol Acarbose equivalents per g of extract.

**Table 6:** Phenolic composition of the optimised *A. subcrenata* Buser extract determined by LC-MS/MS (μg/g extract, mean ± SD, *n* = 3).

Compound	Class	Content (μg/g Extract)
Gallic acid	Hydroxybenzoic acid	196.4 ± 2.620
Protocatechuic acid	Hydroxybenzoic acid	27.87 ± 0.041
2,5-Dihydroxybenzoic acid	Hydroxybenzoic acid	29.69 ± 1.794
*p*-Hydroxybenzoic acid	Hydroxybenzoic acid	7.136 ± 1.036
Vanillic acid	Hydroxybenzoic acid	8.154 ± 1.330
Syringic acid	Hydroxybenzoic acid	0.580 ± 0.013
Aesculetin	Coumarin	6.697 ± 1.086
Quinic acid	Alicyclic acid	152.4 ± 11.530
Chlorogenic acid	Hydroxycinnamic acid	45.32 ± 10.408
Caffeic acid	Hydroxycinnamic acid	23.13 ± 2.661
*p*-Coumaric acid	Hydroxycinnamic acid	50.68 ± 5.851
Ferulic acid	Hydroxycinnamic acid	20.93 ± 4.741
Sinapic acid	Hydroxycinnamic acid	0.903 ± 0.259
Catechin	Flavan-3-ol	161.1 ± 10.33
Quercetin-3-*O*-hexoside	Flavonol glycoside	666.5 ± 92.599
Kaempferol-3-*O*-glucoside	Flavonol glycoside	103.3 ± 22.438
Rutin	Flavonol glycoside	35.61 ± 8.139
Quercitrin	Flavonol glycoside	11.61 ± 1.382
Quercetin	Flavonol aglycone	11.50 ± 1.282
Isorhamnetin	Flavonol aglycone	2.092 ± 0.201
Kaempferol	Flavonol aglycone	1.742 ± 0.157
Luteolin-7-*O*-glucoside	Flavone glycoside	23.56 ± 3.861
Apigenin-7-*O*-glucoside	Flavone glycoside	4.773 ± 0.083
Vitexin	Flavone C-glycoside	0.051 ± 0.005
Luteolin	Flavone aglycone	3.552 ± 0.156
Chrysoeriol	Flavone aglycone	2.625 ± 0.314
Apigenin	Flavone aglycone	1.400 ± 0.196
**Flavanone**		
Naringenin	Flavanone aglycone	0.353 ± 0.092

**Table 7:** Coded levels and experimental values of independent variables for the optimization of MAE of *A. subcrenata*.

Independent Variable	Coded	Level	
		Uncoded	Coded
Ethanol concentration (%)	X_1_	30	−1
		50	0
		70	1
Solid-to-liquid ratio (*w*/*v*)	X_2_	1:20	−1
		1:15	0
		1:10	1
Extraction time (min)	X_3_	10	−1
		20	0
		30	1

## Data Availability

The original contributions presented in the study are included in the article. Further inquiries can be directed to the corresponding author.

## Supplementary Materials

The following supporting information can be downloaded at: https://www.mdpi.com/article/10.3390/plants15142161/s1, Figure S1: Profiles for predicted values and desirability for the responses of *Alchemilla subcrenata* Buser extracts obtained by microwave-assisted extraction, as a function of the coded levels of the independent variables (X1—ethanol concentration, X2—solid-to-solvent ratio, X3—extraction time); Figure S2: Observed-versus-predicted value plots for the twelve response variables (TPC, TFC, DPPH, ABTS, CUPRAC, FRAP, MC, PM, AChE, BChE, tyrosinase, and α-amylase) of *Alchemilla subcrenata* Buser extracts obtained by microwave-assisted extraction, based on the fitted second-order polynomial models; Figure S3: Predicted-versus-residual value plots for the twelve response variables (TPC, TFC, DPPH, ABTS, CUPRAC, FRAP, MC, PM, AChE, BChE, tyrosinase, and α-amylase) of *Alchemilla subcrenata* Buser extracts obtained by microwave-assisted extraction, based on the fitted second-order polynomial models; Table S1: Analysis of variance (ANOVA) for the quadratic models of the responses of *Alchemilla subcrenata* Buser extracts obtained by microwave-assisted extraction, including total phenolic content, total flavonoid content, antioxidant capacity (DPPH, ABTS, CUPRAC, FRAP, metal chelation, and phosphomolybdenum), and enzyme inhibitory activities (AChE, BChE, tyrosinase, and α-amylase); Table S2: Retention times and MS parameters (fragmentor voltage, precursor and product ion *m*/*z*, and collision energy) used for LC-MS/MS quantification of the 44 target phenolic compounds.

## Abbreviations

The following abbreviations are used in this manuscript:

MAE	Microwave-Assisted Extraction
RSM	Response Surface Methodology
LC-MS/MS	Liquid Chromatography-Tandem Mass Spectrometry
HPLC	High-Performance Liquid Chromatography
TPC	Total Phenolic Content
TFC	Total Flavonoid Content
GAE	Gallic Acid Equivalent
RE	Rutin Equivalent
EtOH	Ethanol
BBD	Box–Behnken Design
QE	Quercetin Equivalent
DPPH	2,2-diphenyl-1-picryl-hydrazyl-hydrate
ABTS	2,2′-azino-bis(3-ethylbenzothiazoline-6-sulphonic acid)
CUPRAC	Cupric reducing antioxidant capacity
FRAP	Ferric reducing antioxidant power
MC	Metal chelating assay
PM	Phosphomolybdenum assay
TE	Trolox equivalents
EDTA	Ethylenediaminetetraacetic acid equivalents
AChE	Acetylcholinesterase
BChE	Butyrylcholinesterase
GALAE	Galantamine equivalents
KAE	Kojic acid equivalents
ACAE	Acarbose equivalent
DTNB	5,5′-dithiobis(2-nitrobenzoic acid)
