# Supplementary material for: Unlocking the Bioactive Power of Alchemilla subcrenata Buser via Optimized Green Extraction
Source: Plants (Basel). 2026 Jul 13;15(14):2161. doi: 10.3390/plants15142161 (PMC13416287; doi:10.3390/plants15142161)
Supplement: Supplementary file 1 [file plants-15-02161-s001.zip › plants-4395471-supplementary.pdf]

Supplementary Table S1. Analysis of variance (ANOVA) for the quadratic models of the responses of *Alchemilla subcrenata* Buser extracts obtained by microwave-assisted extraction, including total phenolic content, total flavonoid content, antioxidant capacity (DPPH, ABTS, CUPRAC, FRAP, metal chelation, and phosphomolybdenum), and enzyme inhibitory activities (AChE, BChE, tyrosinase, and α-amylase).

**Response- TPC**	**SS**	**DF**	**MS**	**F-value**	**P-value**
(1)X1 (L)	56.658	1	56.6579	77.7309	0.012622
X1 (Q)	605.040	1	605.0397	830.0740	0.001203
(2)X2 (L)	2.005	1	2.0051	2.7508	0.239067
X2 (Q)	310.997	1	310.9969	426.6670	0.002336
(3)X3 (L)	69.017	1	69.0168	94.6865	0.010397
X3 (Q)	108.267	1	108.2666	148.5345	0.006665
1L by 2L	43.927	1	43.9266	60.2644	0.016192
1L by 3L	63.016	1	63.0160	86.4537	0.011370
2L by 3L	4.260	1	4.2596	5.8438	0.136853
Lack of Fit	177.786	3	59.2620	81.3035	0.012175
Pure Error	1.458	2	0.7289		
Total SS	1454.627	14			
**Response-TF**	**SS**	**DF**	**MS**	**F-value**	**P-value**
(1)X1 (L)	12.6067	1	12.6067	87.609	0.011223
X1 (Q)	176.7585	1	176.7585	1228.364	0.000813
(2)X2 (L)	5.7218	1	5.7218	39.763	0.024239
X2 (Q)	122.5487	1	122.5487	851.639	0.001172
(3)X3 (L)	34.3468	1	34.3468	238.690	0.004163
X3 (Q)	50.7172	1	50.7172	352.454	0.002825
1L by 2L	14.3608	1	14.3608	99.799	0.009872
1L by 3L	107.5629	1	107.5629	747.497	0.001335
2L by 3L	0.0575	1	0.0575	0.400	0.591816
Lack of Fit	53.7269	3	17.9090	124.456	0.007981
Pure Error	0.2878	2	0.1439		
Total SS	586.3894	14			
**Response-DPPH**	**SS**	**DF**	**MS**	**F-value**	**P-value**
(1)X1 (L)	27499.3	1	27499.31	4216.46	0.000237
X1 (Q)	79807.6	1	79807.57	12236.87	0.000082
(2)X2 (L)	1277.6	1	1277.65	195.90	0.005066
X2 (Q)	29560.2	1	29560.21	4532.46	0.000221
(3)X3 (L)	12176.2	1	12176.19	1866.97	0.000535
X3 (Q)	25345.6	1	25345.59	3886.23	0.000257
1L by 2L	8158.9	1	8158.90	1251.00	0.000798
1L by 3L	5507.1	1	5507.11	844.40	0.001182
2L by 3L	11720.0	1	11719.97	1797.02	0.000556
Lack of Fit	17096.8	3	5698.94	873.82	0.001143
Pure Error	13.0	2	6.52		
Total SS	223093.5	14			
**Response-ABTS**	**SS**	**DF**	**MS**	**F-value**	**P-value**

(1)X1 (L)	95329.9	1	95329.9	2444494	0.000000
X1 (Q)	284073.3	1	284073.3	7284343	0.000000
(2)X2 (L)	4369.8	1	4369.8	112053	0.000009
X2 (Q)	199122.8	1	199122.8	5106001	0.000000
(3)X3 (L)	3272.1	1	3272.1	83905	0.000012
X3 (Q)	124662.9	1	124662.9	3196665	0.000000
1L by 2L	1173.8	1	1173.8	30099	0.000033
1L by 3L	14258.3	1	14258.3	365618	0.000003
2L by 3L	41884.2	1	41884.2	1074014	0.000001
Lack of Fit	39127.0	3	13042.3	334437	0.000003
Pure Error	0.1	2	0.0		
Total SS	829614.2	14			
**Response-CUPRAC**	**SS**	**DF**	**MS**	**F-value**	**P-value**
(1)X1 (L)	33902.4	1	33902.44	8576.04	0.000117
X1 (Q)	16090.3	1	16090.34	4070.25	0.000246
(2)X2 (L)	1873.1	1	1873.14	473.83	0.002104
X2 (Q)	44280.9	1	44280.88	11201.40	0.000089
(3)X3 (L)	25945.0	1	25944.98	6563.11	0.000152
X3 (Q)	99711.8	1	99711.77	25223.33	0.000040
1L by 2L	3705.7	1	3705.67	937.39	0.001065
1L by 3L	16778.2	1	16778.16	4244.25	0.000236
2L by 3L	5359.4	1	5359.37	1355.72	0.000737
Lack of Fit	16009.5	3	5336.51	1349.94	0.000740
Pure Error	7.9	2	3.95		
Total SS	276971.0	14			
**Response-FRAP**	**SS**	**DF**	**MS**	**F-value**	**P-value**
(1)X1 (L)	7887.8	1	7887.80	2794.30	0.000358
X1 (Q)	12951.7	1	12951.70	4588.22	0.000218
(2)X2 (L)	8.2	1	8.19	2.90	0.230574
X2 (Q)	45458.6	1	45458.63	16104.01	0.000062
(3)X3 (L)	16098.6	1	16098.63	5703.04	0.000175
X3 (Q)	52749.0	1	52748.99	18686.66	0.000054
1L by 2L	542.1	1	542.14	192.06	0.005166
1L by 3L	8092.2	1	8092.23	2866.72	0.000349
2L by 3L	3226.9	1	3226.87	1143.14	0.000874
Lack of Fit	6175.1	3	2058.38	729.19	0.001370
Pure Error	5.6	2	2.82		
Total SS	161774.9	14			
**Response-MC**	**SS**	**DF**	**MS**	**F-value**	**P-value**
(1)X1 (L)	1.33731	1	1.337315	19.7305	0.047129
X1 (Q)	9.49420	1	9.494199	140.0757	0.007063
(2)X2 (L)	0.49918	1	0.499178	7.3648	0.113189
X2 (Q)	3.77292	1	3.772923	55.6650	0.017495
(3)X3 (L)	0.02284	1	0.022840	0.3370	0.620273

X3 (Q)	0.68518	1	0.685181	10.1090	0.086307
1L by 2L	0.10252	1	0.102516	1.5125	0.343795
1L by 3L	1.27014	1	1.270137	18.7394	0.049440
2L by 3L	0.01817	1	0.018175	0.2681	0.656166
Lack of Fit	8.25920	3	2.753068	40.6183	0.024124
Pure Error	0.13556	2	0.067779		
Total SS	25.48840	14			
**Response-PM**	**SS**	**DF**	**MS**	**F-value**	**P-value**
(1)X1 (L)	0.983426	1	0.983426	482.4973	0.002066
X1 (Q)	0.134359	1	0.134359	65.9202	0.014833
(2)X2 (L)	0.180899	1	0.180899	88.7544	0.011080
X2 (Q)	0.063742	1	0.063742	31.2736	0.030520
(3)X3 (L)	0.106928	1	0.106928	52.4620	0.018533
X3 (Q)	0.150120	1	0.150120	73.6533	0.013307
1L by 2L	0.016677	1	0.016677	8.1821	0.103575
1L by 3L	0.053705	1	0.053705	26.3493	0.035919
2L by 3L	0.077046	1	0.077046	37.8010	0.025449
Lack of Fit	0.101466	3	0.033822	16.5941	0.057372
Pure Error	0.004076	2	0.002038		
Total SS	1.897590	14			
**Response-AChE**	**SS**	**DF**	**MS**	**F-value**	**P-value**
(1)X1 (L)	0.000565	1	0.000565	1.45331	0.351273
X1 (Q)	0.006516	1	0.006516	16.76332	0.054797
(2)X2 (L)	0.003170	1	0.003170	8.15601	0.103857
X2 (Q)	0.001541	1	0.001541	3.96326	0.184762
(3)X3 (L)	0.003501	1	0.003501	9.00709	0.095401
X3 (Q)	0.016352	1	0.016352	42.06819	0.022956
1L by 2L	0.003756	1	0.003756	9.66331	0.089768
1L by 3L	0.010849	1	0.010849	27.91119	0.034011
2L by 3L	0.001031	1	0.001031	2.65255	0.244932
Lack of Fit	0.002260	3	0.000753	1.93831	0.358157
Pure Error	0.000777	2	0.000389		
Total SS	0.051813	14			
**Response-BChE**	**SS**	**DF**	**MS**	**F-value**	**P-value**
(1)X1 (L)	0.090628	1	0.090628	1065.110	0.000938
X1 (Q)	0.028375	1	0.028375	333.484	0.002985
(2)X2 (L)	0.025364	1	0.025364	298.096	0.003338
X2 (Q)	0.251318	1	0.251318	2953.637	0.000338
(3)X3 (L)	0.085166	1	0.085166	1000.919	0.000998
X3 (Q)	0.666403	1	0.666403	7831.957	0.000128
1L by 2L	0.002415	1	0.002415	28.380	0.033477
1L by 3L	0.110244	1	0.110244	1295.648	0.000771
2L by 3L	0.000583	1	0.000583	6.852	0.120190
Lack of Fit	0.185373	3	0.061791	726.204	0.001375

Pure Error	0.000170	2	0.000085		
Total SS	1.523328	14			
**Response-Tyrosinase**	**SS**	**DF**	**MS**	**F-value**	**P-value**
(1)X1 (L)	39.5968	1	39.59676	120.2606	0.008213
X1 (Q)	12.6873	1	12.68730	38.5330	0.024983
(2)X2 (L)	19.3472	1	19.34717	58.7599	0.016596
X2 (Q)	39.1852	1	39.18521	119.0107	0.008298
(3)X3 (L)	42.6066	1	42.60655	129.4018	0.007639
X3 (Q)	5.0125	1	5.01248	15.2236	0.059851
1L by 2L	8.2008	1	8.20079	24.9069	0.037883
1L by 3L	5.1934	1	5.19342	15.7731	0.057944
2L by 3L	3.6778	1	3.67779	11.1699	0.079055
Lack of Fit	9.7212	3	3.24039	9.8415	0.093638
Pure Error	0.6585	2	0.32926		
Total SS	180.1223	14			
**Response-α-amylase**	**SS**	**DF**	**MS**	**F-value**	**P-value**
(1)X1 (L)	0.014514	1	0.014514	369.2482	0.002697
X1 (Q)	0.000003	1	0.000003	0.0704	0.815591
(2)X2 (L)	0.000132	1	0.000132	3.3685	0.207877
X2 (Q)	0.000836	1	0.000836	21.2599	0.043959
(3)X3 (L)	0.000124	1	0.000124	3.1542	0.217715
X3 (Q)	0.000609	1	0.000609	15.4933	0.058899
1L by 2L	0.000872	1	0.000872	22.1936	0.042225
1L by 3L	0.000269	1	0.000269	6.8543	0.120158
2L by 3L	0.000075	1	0.000075	1.9134	0.300757
Lack of Fit	0.000445	3	0.000148	3.7750	0.216468
Pure Error	0.000079	2	0.000039		
Total SS	0.017884	14			

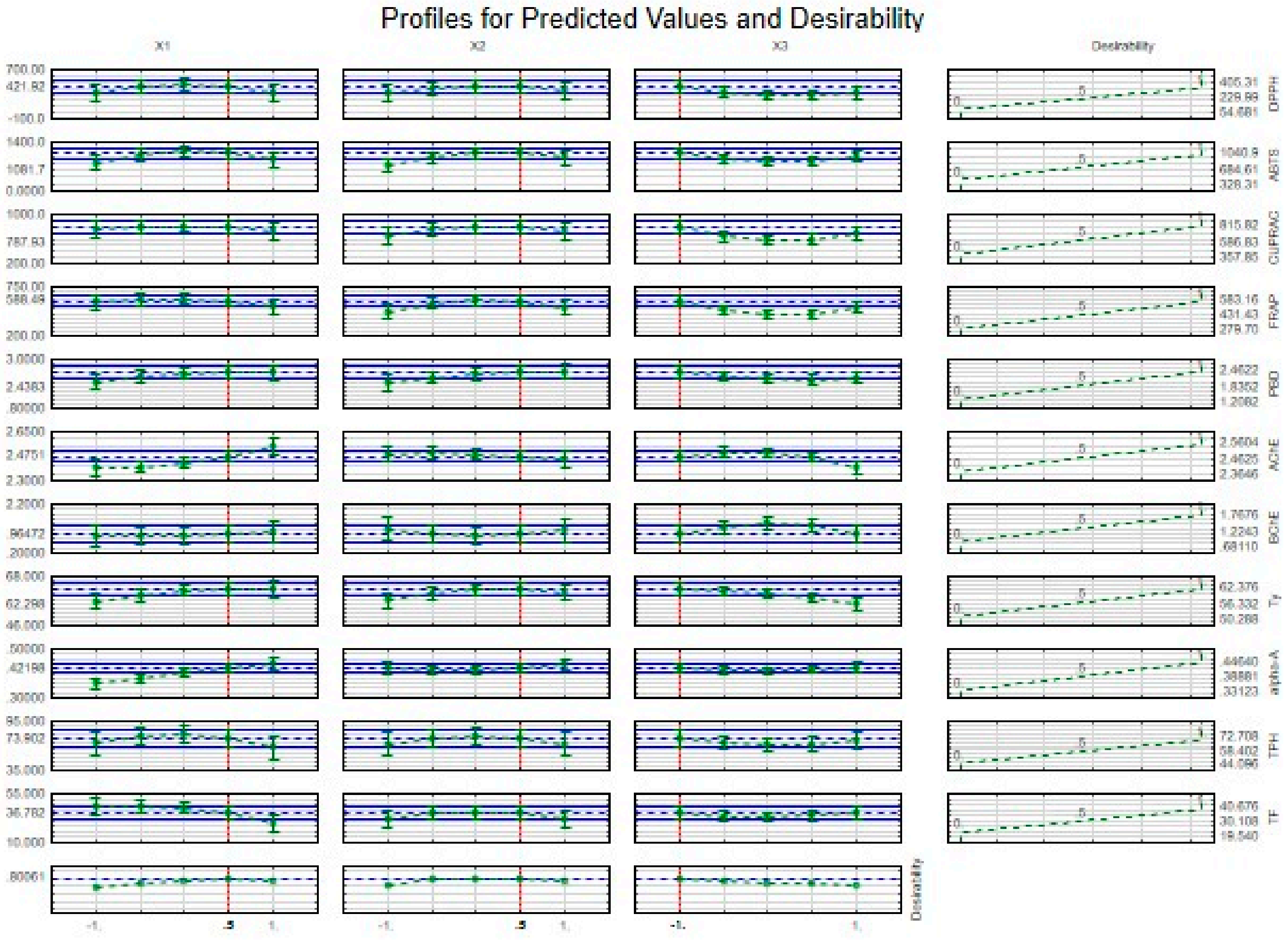

Supplementary Figure S1. Profiles for predicted values and desirability for the responses of *Alchemilla subcrenata* Buser extracts obtained by microwave-assisted extraction, as a function of the coded levels of the independent variables (X1 – ethanol concentration, X2 – solid-to-solvent ratio, X3 – extraction time).

Table S2. Retention times and MS parameters (fragmentor voltage, precursor and product ion *m/z*, and collision energy) used for LC-MS/MS quantification of the 44 target phenolic compounds.

Compound	t_R (min)	Vfragmentor (V)	**Precursor m/z**	**Product m/z**	Vcollision (V)
p-OH-benzoic acid	2.26	80	**137**	**93**	10
Protocatechuic acid	1.63	105	**153**	**109**	9
2,5-Dihydroxybenzoic acid	2.13	100	**153**	**109**	9
p-Coumaric acid	3.52	90	**163**	**119**	9
o-coumaric acid	5.4	100	**163**	**119**	5
Vanillic acid	2.581	100	**167**	**108**	15
Gallic acid	1.23	90	**169**	**125**	10
Aesculetin	2.32	105	**177**	**133**	15
caffeic acid	2.41	100	**179**	**135**	10
Quinic acid	1.08	150	**191**	**85**	20
scopoletin	3.52	80	**191**	**176**	8
Ferulic acid	3.891	90	**193**	**134**	11
Syringic acid	2.685	90	**197**	**182**	7
3,4-Dimethoxycinnamic acid	6.089	110	**207**	**103**	7
sinapinic acid	3. 82	100	**223**	**193**	17
daidzein	7.162	145	**253**	**208**	31
naringenin	7.89	130	**271**	**151**	16
catchine	1.52	150	**289**	**245**	10
Epicatechin	2.12	150	**289**	**245**	10
quercetin	7.74	130	**301**	**151**	15
Isorhamnetin	9.75	160	**315**	**300**	21
Myricetin	5.67	150	**317**	**179**	20
Chlorogenic acid	1.712	100	**353**	**191**	10
vitexin	3.99	200	**431**	**311**	22
Kaem-3-*O*-Glc	5.75	190	**447**	**284**	30
Lut-7-*O*-Glc	4.451	230	**447**	**285**	30
Quercitrin	5.82	190	**447**	**300**	27
Quer-3-*O*-Hexoside	4.64	210	**463**	**300**	30
Rutin	4.68	135	**609**	**300**	42
cinnamic acid	7.91	100	**147**	**103**	5
umbelliferone	3.58	120	**161**	**133**	19
apigenin	9.58	130	**269**	**117**	25
genistein	8.24	145	**269**	**133**	32
baicalein	10.45	165	**269**	**269**	0
luteolin	8.1	135	**285**	**133**	25
Kaempferol	9.295	130	**285**	**285**	0
Chrysoeriol	9.74	125	**299**	**284**	20
Matairesinol	7..41	130	**357**	**122**	24
Secoisolariciresinol	5.85	130	**361**	**165**	26
Api-7-*O*-Glc	5.58	135	**431**	**268**	41
baicalin	6.76	140	**445**	**269**	22
Epigallocatechin gallate	1.72	165	**457**	**169**	16
Amentoflavone	11.93	220	**537**	**375**	35
Apiin	5.4	250	**563**	**269**	36

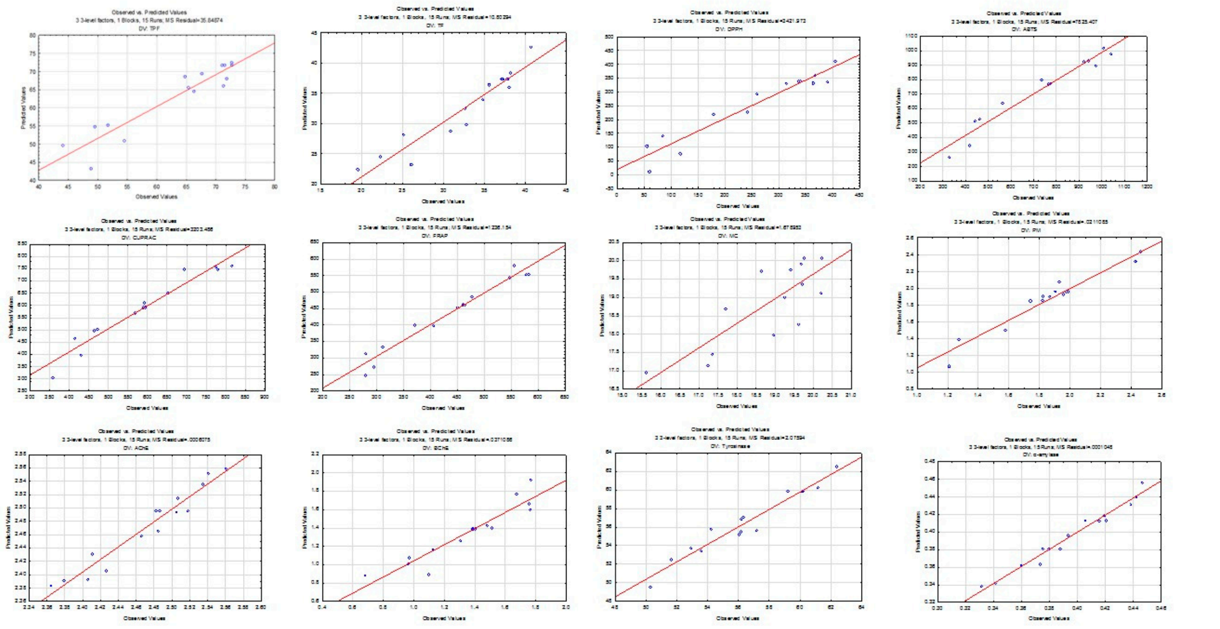

Supplementary Figure S2. Observed-versus-predicted value plots for the twelve response variables (TPC, TFC, DPPH, ABTS, CUPRAC, FRAP, MC, PM, AChE, BChE, tyrosinase, and α-amylase) of *Alchemilla subcrenata* Buser extracts obtained by microwave-assisted extraction, based on the fitted second-order polynomial models.

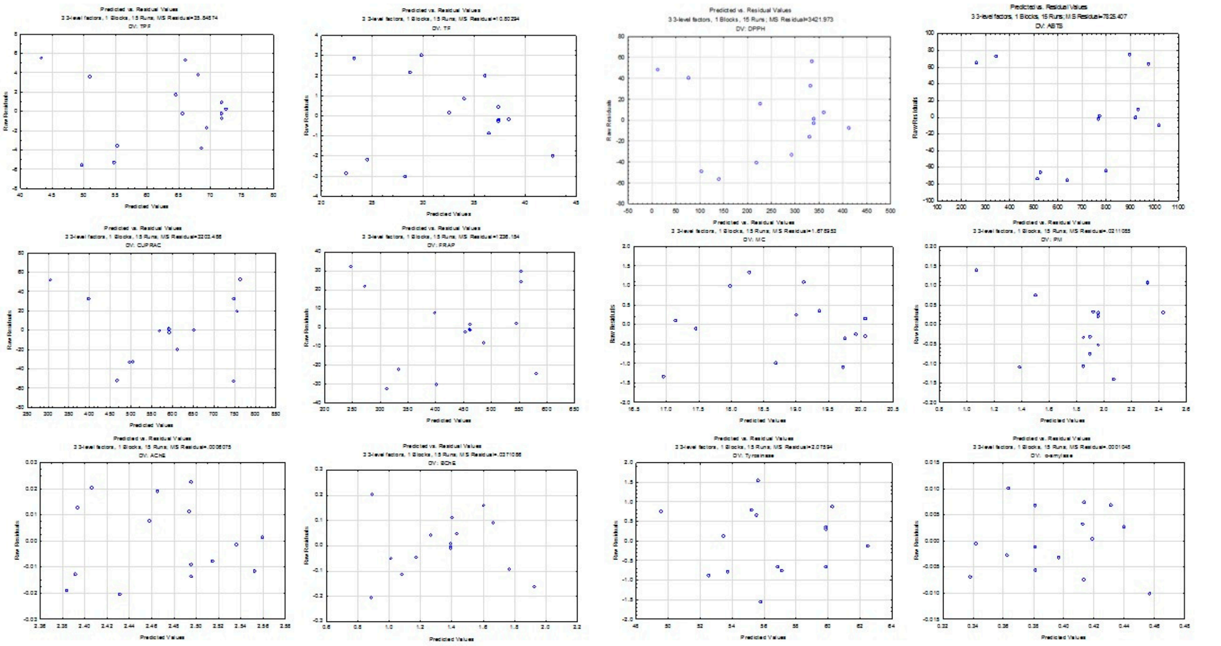

Supplementary Figure S3. Predicted-versus-residual value plots for the twelve response variables (TPC, TFC, DPPH, ABTS, CUPRAC, FRAP, MC, PM, AChE, BChE, tyrosinase, and α-amylase) of *Alchemilla subcrenata* Buser extracts obtained by microwave-assisted extraction, based on the fitted second-order polynomial models.
